# Semiquantitative Decision Tools for FMD Emergency Vaccination Informed by Field Observations and Simulated Outbreak Data

**DOI:** 10.3389/fvets.2017.00043

**Published:** 2017-03-27

**Authors:** Preben William Willeberg, Mohammad AlKhamis, Anette Boklund, Andres M. Perez, Claes Enøe, Tariq Halasa

**Affiliations:** ^1^Department of Diagnostic and Scientific Advice, National Veterinary Institute, Technical University of Denmark, Copenhagen, Denmark; ^2^Environment and Life Sciences Research Center, Kuwait Institute for Scientific Research, Kuwait City, Kuwait; ^3^Department of Veterinary Population Medicine, College of Veterinary Medicine, University of Minnesota, St. Paul, USA

**Keywords:** epidemics, modeling, disease control, risk communication, Foot-and-Mouth Disease

## Abstract

We present two simple, semiquantitative model-based decision tools, based on the principle of first 14 days incidence (FFI). The aim is to estimate the likelihood and the consequences, respectively, of the ultimate size of an ongoing FMD epidemic. The tools allow risk assessors to communicate timely, objectively, and efficiently to risk managers and less technically inclined stakeholders about the potential of introducing FMD suppressive emergency vaccination. To explore the FFI principle with complementary field data, we analyzed the FMD outbreaks in Argentina in 2001, with the 17 affected provinces as the units of observation. Two different vaccination strategies were applied during this extended epidemic. In a series of 5,000 Danish simulated FMD epidemics, the numbers of outbreak herds at day 14 and at the end of the epidemics were estimated under different control strategies. To simplify and optimize the presentation of the resulting data for urgent decisions to be made by the risk managers, we estimated the sensitivity, specificity, as well as the negative and positive predictive values, using a chosen day-14 outbreak number as predictor of the magnitude of the number of remaining post-day-14 outbreaks under a continued basic control strategy. Furthermore, during an ongoing outbreak, the actual cumulative number of detected infected herds at day 14 will be known exactly. Among the number of epidemics lasting >14 days out of the 5,000 simulations under the basic control scenario, we selected those with an assumed accumulated number of detected outbreaks at day 14. The distribution of the estimated number of detected outbreaks at the end of the simulated epidemics minus the number at day 14 was estimated for the epidemics lasting more than 14 days. For comparison, the same was done for identical epidemics (i.e., seeded with the same primary outbreak herds) under a suppressive vaccination scenario. The results indicate that, during the course of an FMD epidemic, simulated likelihood predictions of the remaining epidemic size and of potential benefits of alternative control strategies can be presented to risk managers and other stakeholders in objective and easily communicable ways.

## Introduction

A series of 10 criteria supporting a decision of whether or not to make use of protective emergency FMD-vaccination is listed in Annex X of Council Directive 2003/85/EC (1). These criteria include: “a rapidly rising incidence slope of outbreaks.” A simple, quantitative tool was proposed and documented by Hutber et al. (2), using the “first 14 days incidence” (FFI) of outbreaks in forecasting the duration and the cumulative number of outbreaks at the end using data from 12 regional foci of the UK 2001 FMD epidemic. Thus, according to the abovementioned directive, the FFI might be considered a useful parameter in deciding about the launching of emergency vaccinations in an attempt to lower the total number of outbreaks, as well as to shorten the duration and to lower the losses and costs of an ongoing epidemic.

Modeling the effects of available risk management options during an FMD outbreak in Denmark was undertaken in a recent research project (3). Results and comparisons of simulations of the basic control strategy were compared to different versions of depopulation and vaccination strategies, in terms of their influence on epidemic duration, size, losses, and costs. Simulation results to evaluate the FFI principle were presented by Halasa et al. (4) who introduced the alternative term “first 14 days outbreaks” (FFO).

Decision tools should not only provide scientifically valid results but also have to be transparent and communicable to non-scientists, such as politicians, the media, and the general public to appear trustworthy. Therefore, the technically complex simulation-based results (4) were reformulated as presented here to better allow for communication of the results to the non-scientifically inclined stakeholders. Preliminary results of this work have been presented elsewhere (5–7).

The objectives of this study are as follows:

- To further explore and evaluate the FFI/FFO principle (2), using field data from the FMD outbreaks in Argentina in 2001, with the 17 affected provinces as the units of observation, comprising more than 2,000 outbreaks throughout the country (8). Initially, in-contact herds were vaccinated and imposed with movement control. However, the extensive epidemic was finally controlled by mass vaccination, animal movement control, and active surveillance strategies (9).
- To describe two semiquantitative decision tools based on the FFI/FFO principle as applied to simulated quantitative FMD outbreak data from Denmark (4). These tools are meant for use by risk assessors to document and communicate critical information in a simplified format to risk managers, decision makers, and other stakeholders on the potential benefits and consequences of adding emergency FMD vaccination to the basic control strategy during an emerging FMD epidemic. The tools could also be used as assets to the development and exercise of national FMD contingency plans.

## Materials and Methods

### Argentina

**Figure 1:**
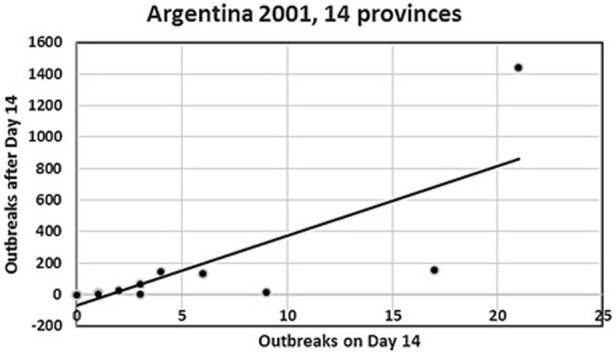
**Plot of number of day 14 outbreaks against number of post-day-14 outbreaks for the Argentina 2001 FMD epidemic from the 14 provinces in the analysis**. The top-right observation is the Buenos Aires province. Four provinces with small numbers of outbreaks are hidden, as they coincide with other close provincial outbreak numbers (see Table 1).

Data from the 2001 FMD outbreaks in the 17 affected provinces were obtained from SENASA, as described previously (8). The number of outbreaks with complete data required for the analyses (2,244 outbreaks or approx. 95% of all recorded outbreaks) are shown in Table 1, where the outbreaks are grouped by time of detection relative to day 14 of the epidemic, as proposed (2). Figure 1 shows a plot of the 14 provincial observations of the relationship between the numbers of accumulated detected outbreaks after day 14 against the accumulated number of outbreaks at day 14. A regression analysis was used to predict number of outbreaks at the end of the epidemic (*C_i_*) using the accumulated number of outbreaks at day 14 as a direct predictor (θ*_i_*) (see Table 1), model I WinBugs version 1.4.3 (10) was used to quantify this relationship through a Bayesian mixed log-linear model, where *C_i_* was assumed to follow a Poisson (λ_i_) process, in which λ is the distribution of the total number of cases in each affected province in 2001. Therefore, the model is formally expressed as: *C_i_* ~ Poisson (λ_i_), log(λ_i_) = β_0_ + β_1_θ_i_ + *U_i_* where β_0_ denotes the model intercept, β_1_ denotes the regression coefficients for θ*_i_*, and *U*_i_ denotes non-structured random effect. *U_i_* is included in the formula to account for lack of independence in the observations due to variables other than θ*_i_*. Non-informative prior distributions of the form *N*~ (0, 0.001) and *N* [0, δ ~ gamma (0.05, 0.005)] were used to model prior knowledge on the value of the regression coefficients. The model was run using 20,000 iterations after burning out the first 1,000 iterations.

**Table 1:** **Detected number of outbreaks at day 14 (θ*_i_*), provincial herd densities (ω*_i_*), and detected and predicted number of outbreaks after day 14 for each affected province**.

Province	Number of outbreaks until day 14 θ*_i_*	(ω*_i_*)	Number of outbreaks after day 14		
			Detected	Predicted	
				*C_i_* model I	*C_i_* model II
Buenos Aires	21	0.2	1,477	1,189	1,049
Catamarca^a^	1	–	0	–	–
Chaco	1	0.11	6	22	16
Cordoba	3	0.36	73	33	68
Corrientes	3	0.08	75	33	21
Entre Rios	4	0.4	159	40	98
Formosa	1	0.11	4	22	16
Jujuy	1	0.03	3	22	11
La Pampa	6	0.07	142	60	35
Mendoza^a^	1	–	0	–	–
Misiones	1	0.3	10	22	36
Rio Negro^a^	2	–	0	–	–
Salta	1	0.04	3	22	12
San Luis	9	0.09	17	108	67
Santa Fe	17	0.2	166	535	491
Santiago del Estero	2	0.06	30	27	16
Tucuman	3	0.12	2	33	24

*^a^Not included in subsequent analyses due to 0 outbreaks after day 14*.

Furthermore, provincial herd densities (ω*_i_*) were included in a second model with θ*_i_* (Table 1, model II), since herd density has been shown to be an important determinant for within-province clustering of FMD herds (8). Confounding of ω*_i_* was evaluated using the method of change in the estimates of β_i_, and best fitting model was assessed based on the smallest value of the deviance information criteria (DIC). Finally, a Kruskal–Wallis test was used to assess the significance of the differences between observed and predicted number of outbreaks at the end of the epidemic.

### Denmark

Danish data were obtained from a series of FMD simulations from models with actual Danish population data on swine, cattle, and sheep herds at the national level and using FFO to designate the cumulative number of outbreaks detected in the first 14-days as a predictor for the size, duration, and costs of the epidemics. The simulation model, the farm data (simulated population), and the simulation study are explained in the next sections.

#### The Simulation Model

The DTU-DADS model (version 0.15) (11) was further updated (to version 0.16) and used to obtain the simulation data for the analysis in this study. The updates included updating the modeling of the local spread and adjusting the code to correct a coding error. For the local spread, in the earlier version of the model, the probability of infection varied depending on distance from the infectious herds in a maximum of 3 km zone (3, 11). In the current version, the disease could spread locally depending on distance from the infectious herd in the same way as modeled earlier, but depending on time from the start of the infectiousness of the infectious herd using similar probabilities (12).

##### The Farm Data

The data consisted of all cattle, swine, sheep, and goat herds that are registered in the Central Herd Register (CHR) of Denmark in the period from first October 2006 until 30th September 2007. During this period, there were 23,550, 11,473, and 15,830 cattle, swine, and sheep/goat herds, respectively. The data included information about the identification number, the UTM coordinates, the number of animals, and the rate of animal movements per day for each herd. While cattle herds were divided into milking and non-milking herds, sheep and goats were grouped into one category, while swine herds were split into 19 types (13). When a farm included several animal species, each species was given a different ID and set as a different herd on the same location and with the same CHR number. Further details about the study population and model input parameters can be found elsewhere (3, 11).

#### The Simulation Study

##### Modeling Virus Spread

Spread of infection between herds was simulated through seven spread mechanisms: (1) direct animal movement between herds; (2) abattoir trucks; (3) milk tankers; (4) veterinarians, artificial inseminators, and/or a milk controllers (medium risk contact); (5) visitors, feedstuff, and/or rendering trucks (low risk contact); (6) markets; and (7) local spread (11, 14).

The virus spread *via* animal movements and abattoir contacts was simulated based on the rate of movements/contacts per day calculated from actual movement data. For abattoir contacts, an additional parameter representing the number of herds that will be contacted by the abattoir truck on its way to the abattoir following its contact to the infectious herd was included based on the type of the infectious herd. Virus spread *via* medium and low risk contacts was simulated using the daily frequency of contact between herds *via* these routes. Virus spread *via* milk tank was possible only from a milking to another milking herd using the daily frequency of milk pickup from the dairy herds. Virus spread *via* markets was possible initially between cattle herds as markets in Denmark are restricted to cattle only. From markets, the virus could spread to susceptible herds due to movement of animals, people, and/or vehicles (11).

##### Modeling Disease Detection

An infected herd could be detected in one of the three mechanisms, namely: first detection, basic detection, and detection following surveillance or tracing. First detection reflected the detection of the disease in the country (the index case/outbreak). This occurred following a specific number of days after the introduction of infection. A PERT distribution was used to determine the day of first detection following virus introduction. The minimum, mode, and maximum values were 18, 21, and 23 days, respectively. Basic detection reflected the farmers’ awareness of a problem within their herds and hence calling the veterinarian, while detection through surveillance or tracing occurred following a visit by the veterinary authorities. The probabilities of detection using the last two detection mechanisms were dependent on the type of the herd (11).

##### Modeling Disease Control

Once the first infected herd is detected, a set of control actions are enforced as explained earlier (11). These actions include (1) depopulation, cleaning, and disinfection of all detected herds, (2) the implementation of a 3-km protection zone and a 15-km surveillance zone around each detected herd, (3) all susceptible herds within the zones are surveyed and animal movements and contacts between herds are restricted within the zones, (4) forward and backward tracing of animal movements and contacts to and from detected herds, and (5) the implementation of a 3-day national stand still on animal movements. In addition, herds that received animals from detected herds were depopulated (11).

Extra control strategies, including preemptive depopulation or suppressive vaccination, were adopted in separate scenarios. When implemented, herds with susceptible animals within a 1-km radius around newly detected herds were subjected to the extra control. The extra control strategy was initiated 14 days after first detection.

##### Initiation of Simulation and Model Run

The simulation started with the model loading the input data and, thereafter, selecting the primary outbreak herd, which is the first infected herd in the epidemic. About 5,000 cattle herds were selected randomly as potential primary outbreak herds to initiate disease spread. Earlier results have shown that epidemics initiated in cattle herds would provide larger spread than epidemics initiated in other species (14), reflecting a worse-case scenario. Each primary outbreak herd initiated an epidemic once, resulting in outbreak data from 5,000 epidemics.

The outcomes of the model included epidemic duration (number of days between first detection and the culling of the last detected herd), number of infected herds, number of depopulated or vaccinated herds, and the total costs of the epidemics. The total costs were calculated as direct cost and export losses (11).

#### The Decision Tools

To simplify the presentation of pros and cons of vaccination to all stakeholders and to enable the urgent decisions to be made by the risk managers during the course of an ongoing FMD epidemic, a two-step methodology based on the FFO principle was applied in presenting the Danish simulation outcomes.

##### Quantifying Uncertainty (Predictive Values) of Estimating the Likelihood of a “Catastrophic” Epidemic

During an ongoing national FMD epidemic, the actual cumulative number of outbreaks at day 14 will have a given observed value, e.g., 15 herds (i.e., FFO = 15). Data from the simulated epidemics lasting more than 14 days were distributed among the cells of a two-by-two table based on a selected cutoff value for both the independent (i.e., FFO = 15) and of the dependent variables (a chosen “catastrophic” number of post-day-14 outbreaks, e.g., 50 or 100). This enables estimation of sensitivity, specificity, and negative and positive predictive values describing the association between the observed FFO value and a “catastrophic” epidemic in terms of the cumulative number of outbreaks occurring after day 14.

The estimation procedure described above was performed using simulated data for the basic control strategy throughout 5,000 simulated epidemics in Denmark.

If applied as part of an exercise to update FMD contingency plans, a series of alternative FFO and “catastrophic” simulated outbreak numbers post-day-14 might be explored for the comparative control strategies.

##### Quantifying the Consequences (Expected Benefits) in Terms of the Number of Prevented “Catastrophic” Outbreaks when Changing to a Vaccination Strategy at Day 14 during the Epidemic

The frequency distribution of the observed number of total cumulative outbreaks for the series of simulated epidemics with the observed fixed FFO-value were compared for the basic control strategy and the vaccination strategy, with both strategies applied to the same set of simulated epidemics in terms of the seeded primary outbreak herds. The benefit of changing from basic control to emergency vaccination can be estimated by comparing the number and proportion of “catastrophic” epidemics expected in the basic with those in the vaccination simulations.

## Results

### The Argentinian Epidemic

Table 2 summarizes the posterior estimates for the two regression models. While θ*_i_* is a significant predictor for C_i_ by itself, ω*_i_* is an important confounder based on the method of the change in the posterior estimate (i.e., the constant’s coefficient changed by 27%), a significant predictor (97.5% CI), and substantially improved the fit of the model (smallest DIC value).

**Table 2:** **Regression model parameters estimated from the Argentina 2001 epidemic data**.

Regression coefficients	Posterior estimates	SD	Monte Carlo error	97.5% CI^a^	Deviance information criteria
**Model I**					
β_0_	2.89	0.061	<0.001	(2.77, 3.00)	1,199.61
β_1_θ*_i_*	0.20	0.003	<0.001	(0.19, 0.21)	
**Model II**					
β_0_	2.11	0.333	<0.001	(1.92, 2.30)	934.61
β_1_θ*_i_*	0.19	0.003	<0.001	(0.19, 0.21)	
β_2_ω*_i_*	4.28	0.096	<0.001	(3.63, 4.93)	

*^a^97.5% credible interval*.

The observed and predicted number of outbreaks for models I and II are summarized in Table 2. No significant differences were identified between the number of observed and the number of predicted outbreaks at the end of the epidemic for models I and II (Kruskal–Wallis *p*-value >0.54).

### Descriptive Results of the Danish Simulations

Table 3 shows the overall descriptive results from the simulation study, using the basic control, preemptive depopulation, and suppressive vaccination scenarios. For instance, using the median basic scenario, epidemic duration is predicted to be 36 days (5th and 95th percentiles 2–128 days), resulting in 22 infected herds (5th and 95th percentiles 2–145 herds), and a total loss of €869 million (5th and 95th percentiles €703–€1,434 million).

**Table 3:** **Summary of the results of 5,000 simulated Danish FMD epidemics all starting in cattle herds; median and 5–95% CI**.

Control strategy	Epidemic duration	Infected herds	Culled herds	Vaccinated herds	Total costs (€ million)
Basic control throughout	36 (2–128)	22 (2–145)	22 (2–145)	0	869 (703–1,434)
Preemptive depopulation^a^	25 (2–50)	19 (2–67)	35 (2–150)	0	807 (703–994)
Suppressive vaccination^a^	36 (2–98)	22 (2–105)	22 (2–105)	24 (0–184)	863 (703–1,284)

*^a^Change from basic control after day 14*.

**Figure 2:**
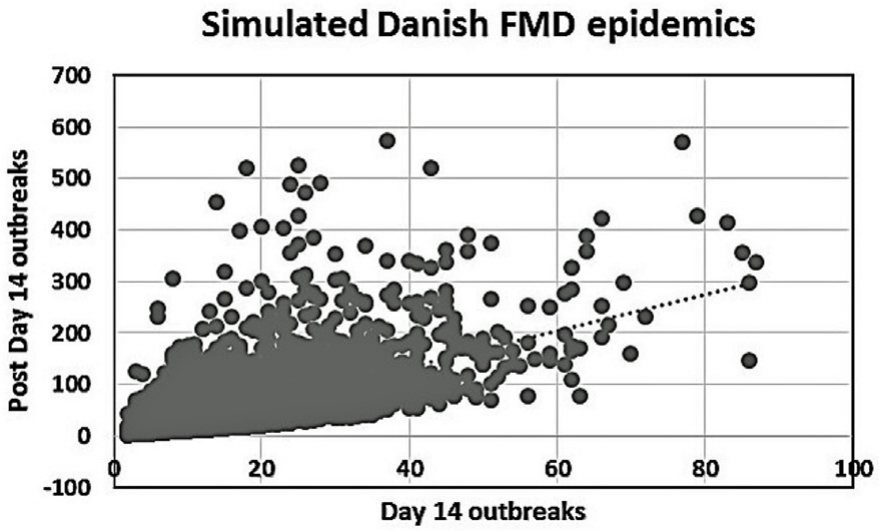
**Plot of the number of day 14 outbreaks against the number of post-day 14 outbreaks for the 4,092 simulated Danish FMD epidemics lasting more than 14 days**.

Among the group of 5,000 simulations specifically considered here, 4,092 epidemics lasted >14 days. Figure 2 shows a plot of these simulated epidemics with their accumulated number of outbreaks occurring after day 14 until the end of the epidemics, against the accumulated number of outbreaks at day 14.

### Quantifying Predictive Values Based on the FFO Principle

For a set of chosen cutoff values [i.e., FFO < 15 vs. FFO ≥ 15 outbreaks and <50 vs. ≥50 outbreaks recorded after day 14 (Table 4)], the negative predictive value (NPV) is 93% and the positive predictive value (PPV) is 33%. This would mean a 7% probability of an epidemic with <15 outbreaks at day 14 becoming a “catastrophic epidemic” of ≥50 subsequent outbreaks, if the basic strategy was continued, while there would be a 33% probability that an epidemic with ≥15 outbreaks at day14 would turn out to be a” catastrophic epidemic” of ≥50 subsequent outbreaks with no change in strategy.

**Table 4:** **Danish-simulated FMD epidemics lasting more than 14 days: specificity, sensitivity, negative predictive value (NPV), and positive predictive value (PPV) for two alternative combinations (A and B) of the presumed observed cumulative outbreak size on day 14, and the subsequent cumulative outbreak size until the end of the epidemic**.

**A: more or less than 50 outbreaks expected subsequently**				

**Outbreaks on day 14**	**Outbreaks after day 14**			
	**<50**	**≥50**	**Total**	

<15	2,009	156	2,165	NPV = 93%
≥15	1,284	643	1,927	PPV = 33%
Total	3,293	799	4,092	*p*(≥50) = 20%
	Sp = 61%	Se = 80%		

**B: more or less than 100 outbreaks expected subsequently**				

**Outbreaks on day 14**	**Outbreaks after day 14**			
	**<100**	**≥100**	**Total**	

<15	2,092	73	2,165	NPV = 97%
≥15	1,719	208	1,927	PPV = 11%
Total	3,811	281	4,092	*p*(≥100) = 7%
	Sp = 55%	Se = 74%		

Changing the cutoff value for “catastrophic” epidemics to ≥100 outbreaks changes the NPV to 97% (Table 4). This means that if <15 infected herds have been detected up until day 14, there would be an estimated probability of just 3% that the ongoing epidemic under a continued basic control strategy would result in a cumulative number of outbreaks of ≥100. The PPV, however, is estimated at only 11%, which is explainable by the relatively low probability of “catastrophic” epidemics of ≥100 outbreaks among the simulated outcomes (*p* = 7%).

### Quantifying the Expected Benefit of Changing to a Vaccination Strategy during an Epidemic

**Figure 3:**
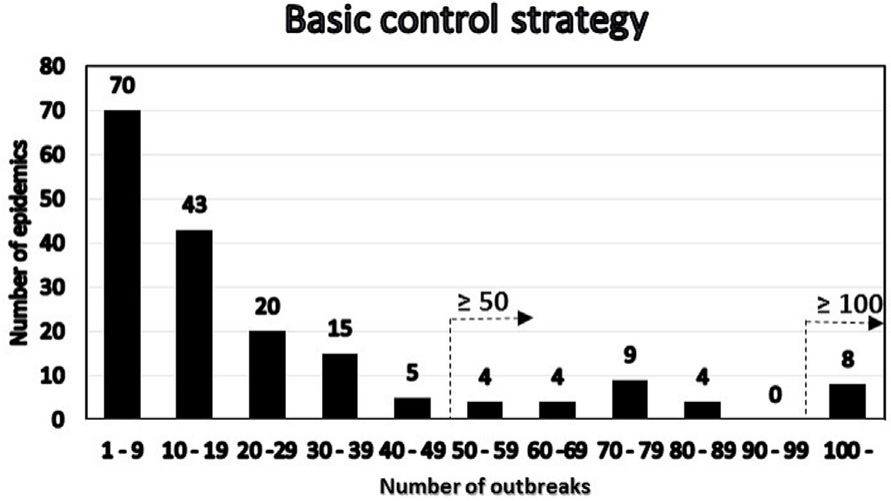
**Distribution under the basic control scenario of 182 FMD-epidemics with 15 detected herds at day 14 by number of post-day 14 outbreaks**. The two alternative definitions of “catastrophic” epidemics in terms of number of outbreaks are indicated.

**Figure 4:**
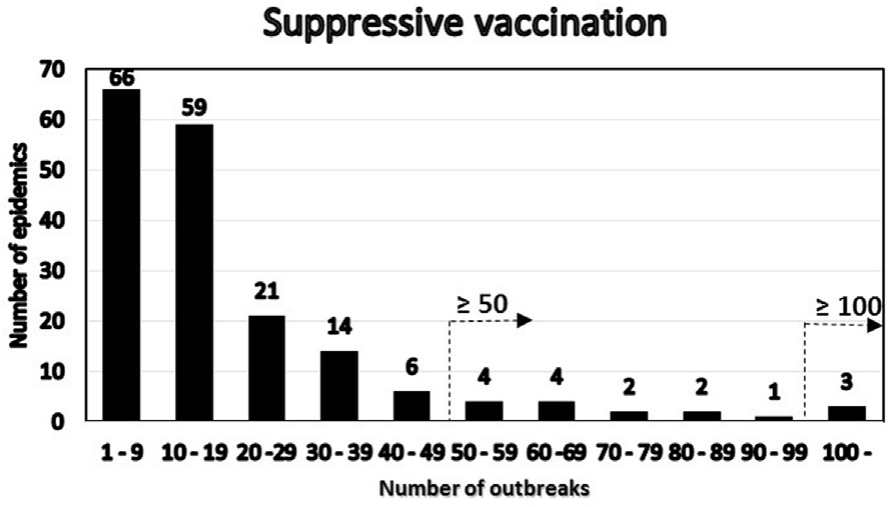
**Distribution under the emergency vaccination scenario of the 182 epidemics with 15 detected herds at day 14 by number of post-day 14 outbreaks**. The two alternative definitions of “catastrophic” epidemics in terms of number of outbreaks are indicated.

Among the simulated epidemics lasting 14 days or more, all the simulations with a cumulative number of outbreaks equal to 15 were chosen (i.e., assuming that in an ongoing field epidemic, FFO = 15), resulting in 182 epidemics, which were further analyzed. The distribution of the number of outbreaks at the end of the epidemics minus the FFO-value of 15 was determined (Figure 3). The distribution of the number of outbreaks under the basic control scenario is compared to that under a suppressive vaccination scenario for the same 182 epidemics, i.e., using the same primary outbreak herds as in the basic control scenario (Figure 4). Of the eight “catastrophic epidemics” with ≥100 outbreaks after day 14 in the basic scenario, 5 (63%) were predicted to be spared by applying emergency vaccination. Using 50 outbreaks as the cutoff, 13 out of the 29 (i.e., 45%) of these “catastrophic epidemics” were predicted to be spared by vaccination, see Figures 3 and 4.

## Discussion

For the Argentina 2001 epidemic, the median herd disease reproduction ratio decreased significantly from 2.4 (before the epidemic was officially recognized) to 1.2 during the mass-vaccination campaign and <1 following the mass-vaccination campaign (9). This is consistent with our finding of the agreement with the FFO principle for this epidemic, although once the index outbreak was detected, control activities were applied including both emergency vaccination of in-contact-herds, and subsequently mass vaccination, which started late and lasted for a long period due the extended area and large numbers of herds to be covered (8, 9). One would generally expect a marked decrease in the number of outbreaks following vaccination, but in this case, the initial intervention probably was of insufficient magnitude to effectively control the spread, resulting in a substantial epidemic tail-end in terms of number of outbreaks, duration, and geographical coverage. Had it been possible to monitor the early part of the epidemic and to apply the FFI/FFO tools described here, i.e., to act on day 14 of the epidemic, it might have led to a smaller epidemic and a faster recovery. It is worth noting that the magnitude of the association between the accumulated number of outbreaks at day 14 and the subsequent number of cases in each affected province, as indicated by the value of the regression coefficient (Table 2), is influenced by the presence of the large outbreak contributions from the Buenos Aires province (see Figure 1). However, the nature of the association, as indicated by the positive sign of the regression coefficient and its significance (see 97.5% CI, Table 2) remains (data not shown). For that reason, we conclude that the model was robust to the inclusion or exclusion of Buenos Aires in the analysis. Inclusion is justified, however, by the biological and economical significance of the 67% of the total number of outbreaks, which this province accounted for.

A high degree of variation is seen in the Danish simulation results (Figure 2). This is likely due to the Danish data describing 5,000 different simulated epidemics, while both the British (2) and the Argentinean data considered here were concerned with regional variations within individual extended field epidemics. Our main finding as shown in Figures 3 and 4, favoring vaccination over continued basic control when aiming to avoid catastrophic epidemics, is consistent with the overall results of the Danish simulation study (4). As can be seen from Table 3, on average, the alternative control strategies do not differ much; however, the extreme upper range values tend to be lower for the vaccination and cull strategies than for the basic control strategy. The relatively low positive predictive values (Tables 4) of course influence the average benefit/cost ratio of implementing a vaccination strategy based on this procedure, as many such vaccination campaigns apparently may be wasted, since by far most epidemics would entail <50 outbreaks with just the basic control strategy. This might indicate that the basic control strategy could be continued with a reasonably high degree of confidence. So here, also the PPV indicates a limited effect to be expected from a change of strategy toward vaccination. Apparently, a cutoff value of 100 predicted outbreaks to be used for vaccination considerations may be too high to yield useful decision criteria, because only a small percentage (here 7%) of simulated outbreaks reach that level under the basic control strategy. Such information would be valuable to note, when using simulations as part of FMD contingency planning and exercising. The benefits of possibly reducing the actual number of outbreaks within “non-catastrophic” epidemics due to vaccination are, however, not taken into account in these estimates.

The added economic costs introduced by applying FMD vaccination should be considered when setting the cutoff for what would be a “catastrophic epidemic” in terms of the number of outbreaks. Implementing vaccination in a control strategy by itself might be very costly, e.g., due to a lengthier trade ban for Danish animals and products on the export markets (3). Thus, risk managers might tolerate up to a moderate likelihood of a high number of outbreaks in order to avoid these economic consequences of vaccination. However, if the decision tool predicts vaccination to spare a relative large number of outbreaks, the added costs may appear acceptable, also considering the welfare benefits of a limited culling after implementation of suppressive vaccination strategy.

Along with the aspects of risk assessment and risk management discussed above, risk communication is an equally important part of an FMD risk analysis in the face of an ongoing epidemic (15). The interactions of these three components are nowhere more critical than in the initial phases of a national FMD epidemic, when alternative control strategies must be considered. Fast and reliable assessment of the likelihood and consequences of spread and the continuous evaluation and selection of optimal management and control measures should be supported by timely, robust, and transparent communication among risk assessors, risk managers, and other stakeholders. Only then may urgent and critically important decisions be properly understood and accepted. Emergency vaccination should be considered, if the anticipated cumulative size of the epidemic under a continued basic control strategy appears alarming and if a sufficient reduction can be expected in the magnitude and duration of the epidemic to justify the estimated additional direct and indirect control costs incurred by vaccination (3).

Several FMD epidemics affected Europe and South America in 2001 and the control strategies applied have been discussed extensively (8, 9), major obstacles to effective prevention, detection, and control of FMD have been identified and the role of disease models in animal health emergency preparedness has been highlighted (16–18). In particular, the following statements characterize the situation facing authorities when it comes to decisions on the potential use of emergency vaccination during an FMD epidemic:

- The decisions about if and how to vaccinate during a major FMD epidemic are complex (9).
- The choice of whether or not to apply emergency vaccination is probably the most difficult decision facing the authorities when disease breaks out in an erstwhile FMD-free country. Effective computational models should be actively financed for a range of outbreak scenarios to assist objective decision-making and minimize bureaucratic delays in vaccine application (19).
- There is a need for better analytical tools to support decisions for FMD control (20).

When future epidemics occur, scientific and political debate will rise again regarding the merits of vaccination, with many technical, logistical, economic, political, cultural, and historical facts affecting the decision. Generally, vaccination decisions have to be made quickly and will be influenced greatly by previous experiences, but because large FMD epidemics are extremely rare events, the opportunities to directly assess the effects of control strategies are very limited (9). Therefore, effective computational models should be made available for a range of outbreak scenarios to assist objective decision-making and minimize bureaucratic delays in vaccine application, and continued efforts are required to develop robust models for use during outbreaks in FMD-free countries (19, 21). Comparison of the pros and cons of alternative control strategies has been the aim of numerous simulation modeling studies, as recently reviewed and discussed (21, 22). The special importance of communicating output results from modeling tools to decision makers has been highlighted by the European Commission for the control of Foot-and-Mouth Disease—EuFMD (23):

- Member states should consider the use of modeling tools as decision-making aids, while ensuring that the output of such models are clearly understood by decision makers with respect to uncertainty and sensitivity.

The results presented here indicate that, in the context of a decision-making aid, choice of control strategy and predictions of epidemic consequences based on the cumulative number of outbreaks detected by day 14 would be useful. Furthermore, results from simulation models comparing alternative control strategies can be documented and communicated to risk managers and stakeholders in simple ways, which seem appropriate in urgently informing decisions about whether or not to implement changes, such as deployment of emergency vaccination.

## Author Contributions

PW developed the extension to the FFO concept of the two decision support tools and completed the initial analyses on the Danish model simulation data to document their applicability. CE contributed to the initial conceptual discussions of the design of the study and participated in acquisition of the Danish data for the simulations. AB and TH provided these data and results from their model simulation studies. AP provided access to the Argentina FMD epidemic data, and MA provided the statistical analyses of these data. PW drafted the initial draft manuscript, and all the co-authors reviewed the work critically, suggested revisions, and finally approved the version to be published.

## Conflict of Interest Statement

The authors declare that the research was conducted in the absence of any commercial or financial relationships that could be construed as a potential conflict of interest.
